# Research on Bearing Fault Diagnosis Based on VMD-RCMWPE Feature Extraction and WOA-SVM-Optimized Multidataset Fusion

**DOI:** 10.3390/s25165139

**Published:** 2025-08-19

**Authors:** Shouda Wang, Chenglong Wang, Youwei Lian, Bin Luo

**Affiliations:** National Engineering Research Center for Technology and Equipment of Green Coating, Lanzhou Jiaotong University, Lanzhou 730070, China; 12232068@stu.lzjtu.edu.cn (S.W.); 12232072@stu.lzjtu.edu.cn (Y.L.); 12232074@stu.lzjtu.edu.cn (B.L.)

**Keywords:** fault detection, WOA, SVM, VMD, RCMWPE

## Abstract

Bearings are critical components whose failures in industrial machinery can lead to catastrophic breakdowns and costly downtime; yet, accurate early-stage diagnosis remains challenging due to the non-stationary, nonlinear nature of vibration signals and noise interference. This study proposes a multidataset-integrated bearing fault diagnosis methodology incorporating variational mode decomposition (VMD), refined composite multiscale weighted permutation entropy (RCMWPE) feature extraction, and whale optimization algorithm (WOA)-optimized support vector machine (SVM). Addressing the non-stationary and nonlinear characteristics of bearing vibration signals, raw signals are first decomposed via VMD to effectively separate intrinsic mode functions (IMFs) carrying distinct frequency components. Subsequently, RCMWPE features are extracted from each IMF component to construct high-dimensional feature vectors. To address visualization challenges and mitigate feature redundancy, the t-distributed stochastic neighbor embedding (t-SNE) algorithm is employed for dimensionality reduction. Finally, WOA optimizes critical SVM parameters to establish an efficient fault classification model. The methodology is validated on two public bearing datasets: PRONOSTIA and CWRU. For four-class fault diagnosis on the PRONOSTIA dataset, the model achieves 96.5% accuracy. Extended to ten-class diagnosis on the CWRU dataset, accuracy reaches 99.67%. Experimental results demonstrate that the proposed method exhibits exceptional fault identification capability, robustness, and generalization performance across diverse datasets and complex fault modes. This approach offers an effective technical pathway for early bearing fault warning and maintenance decision making.

## 1. Introduction

Bearings serve as critical components in modern industrial machinery, directly influencing system stability, efficiency, and operational safety. Prolonged service in high-load, high-speed environments such as wind turbines, high-speed trains, and aerospace systems inevitably induces bearing failure modes including wear, fatigue, and cracks. These failures not only trigger unplanned downtime with significant economic losses but may escalate into catastrophic safety incidents. Consequently, developing precise early-stage bearing fault diagnosis technologies holds substantial engineering value for ensuring continuous production, optimizing maintenance strategies, and enhancing equipment reliability. Traditional diagnostic approaches relying on time-domain statistical features, frequency-domain Fourier analysis, and time-frequency transformations exhibit inherent limitations when processing non-stationary, nonlinear vibration signals, particularly demonstrating inadequate robustness for incipient weak faults under strong noise interference. Recent advances in artificial intelligence have enabled machine learning algorithms like support vector machines and artificial neural networks to show significant potential in fault classification. For signal processing, while empirical mode decomposition adaptively decomposes non-stationary signals into intrinsic mode functions, it suffers from modal aliasing. Ensemble and complete ensemble empirical mode decomposition partially address this limitation through Gaussian white noise-assisted ensemble averaging, whereas variational mode decomposition reformulates decomposition as a variational optimization problem, offering theoretically rigorous foundations with superior noise immunity and modal separation efficacy. Regarding feature extraction, entropy-based complexity metrics are particularly vital. Permutation entropy quantifies signal complexity through ordinal pattern probability; multiscale permutation entropy extends analysis across temporal scales; weighted permutation entropy and multiscale weighted permutation entropy enhance local feature representation via weighting factors; refined composite multiscale weighted permutation entropy further integrates multiscale analysis, weighted entropy, and composite refinement to significantly boost feature robustness and noise immunity. For classifier construction, support vector machines excel in small-sample nonlinear classification yet require careful parameter optimization. Intelligent optimization techniques including whale optimization algorithm, particle swarm optimization, and genetic algorithms effectively address this challenge. To handle high-dimensional features, t-distributed stochastic neighbor embedding enables nonlinear dimensionality reduction while preserving local and global data structures, providing critical support for model visualization and construction. Current research on fault diagnosis for wind turbine gearbox bearings primarily encompasses three methodological domains: feature extraction, pattern recognition, and signal processing techniques. Regarding signal preprocessing, empirical mode decomposition (EMD) [1,2,3,4], ensemble empirical mode decomposition (EEMD) [5,6], and variational mode decomposition (VMD) [7,8,9,10,11,12] are extensively applied to enhance signal-to-noise ratio, mitigate noise interference, and prevent mode mixing. Within feature extraction, information entropy-based approaches [13,14,15] and deep learning methodologies [16,17,18,19] have been widely investigated and implemented by researchers. Addressing classifier parameter selection under conditions of limited sample size and noise contamination, where improving accuracy and efficiency is paramount, support vector machine (SVM) algorithms [20,21,22] effectively fulfill classification optimization requirements, achieving intelligent classification while enhancing both diagnostic efficiency and precision.

Numerous scholars have contributed significantly to research on wind turbine bearing fault diagnosis. Maohua Xiao et al. [23] demonstrated that introducing the improved complete ensemble empirical mode decomposition with adaptive noise (ICEEMDAN) method yielded effective dimensionality reduction; combined with information entropy algorithms, it efficiently separated bearing vibration signal features for identifying different vibration signals. Runze Qi et al. [24] applied ICEEMDAN to dimensionality reduction of transformer mechanical fault signals, finding it enhanced reduction effectiveness, effectively suppressed noise signals, and yielded purer fault feature data. Xiao Yang et al. [25] investigated cross-domain fault diagnosis using refined composite multiscale weighted permutation entropy (RCZMWPE), which enhanced noise resistance, avoided information loss, and captured weak fault features through refined composite multiscale weighted calculation of minimally analyzed coarse-grained sequences. Wei Sun et al. [26] integrated the whale optimization algorithm (WOA) with support vector machine (SVM) to study partial discharge faults in power plants, building virtual models, training data, and identifying fault types, discovering the significant impact of improved WOA (IWOA) on VMD and SVM parameters, with the IWOA-VMD-SVM combination markedly improving accuracy and operational speed. Bing Wang et al. [27] proposed combining a whale swarm algorithm-optimized multilayer SVM with multiscale Kolmogorov entropy for rolling bearing fault signals, achieving 97.8% diagnostic accuracy. Zhen Wang et al. [28] optimized VMD and t-distributed stochastic neighbor embedding (t-SNE), achieving 100% accuracy in parallel-axis gearbox fault diagnosis. Wei He et al. [29] combined singular value decomposition (SVD), t-SNE, and SVM, developing a novel feature extraction path and parametric t-SNE to eliminate irrelevant information and enhance fault severity separability through nonlinear projection. Jiawei Liu et al. [30] combined deep learning with t-SNE for sequential fault diagnosis in proton exchange membrane fuel cell water management subsystems, achieving 96.88% accuracy and broadening the application scope of this combined diagnostic approach. Smith et al. [31] proposed the local mean decomposition (LMD) method. Ma, J et al. [32] proposed the modified VMD and Teager energy operator (MVMD-TEO) method, which autonomously determines the VMD mode number to extract incipient fault features of bearings. Yang et al. [33] determined VMD parameters through computation of central frequency components and envelope spectrum energy ratios between different components. Li, J. et al. [34] proposed an estimation approach for the quadratic penalty term. This method analyzed the spectral distribution patterns of bearing vibration signals, ultimately enabling adaptive determination of the parameters. LI et al. [35] employed a deeply stacked least squares support vector machine (LSSVM) algorithm to acquire intrinsic fault characteristics of rolling bearings through adaptive feature extraction. Ding et al. [36] employed a genetically variant particle swarm optimization (GVPSO) algorithm to determine the decomposition mode number and penalty factor of VMD. Gear fault signals were decomposed into multiple intrinsic mode functions (IMFs), followed by calculation of the sample entropy for each IMF as feature values. These features were subsequently input into a probabilistic neural network (PNN) to achieve precise classification of gear faults. M Nazari et al. [37] proposed a successive variational mode decomposition (SVMD) method. This approach achieved modal decomposition by imposing a series of constraints on the VMD optimization problem, eliminating the need for presetting the number of decomposition modes *k*. It significantly reduced computational complexity, adaptively partitioned the frequency domain and exhibited greater robustness to initializations of modal center frequencies. Yan et al. [20] employed particle swarm optimization (PSO)-tuned SVM to achieve recognition of multiple fault states in rolling bearings. Song et al. [38] utilized wavelet packet threshold denoising combined with a BP neural network to enhance the quality of bearing fault signals and achieve efficient diagnosis. Current research efforts focus on four primary technical routes: deep learning, time-frequency analysis, information entropy, and traditional machine learning. Deep learning relies heavily on massive labeled data, incurs high training costs, involves complex models, and exhibits limited generalization under small samples. Despite the widespread adoption of signal processing techniques (e.g., EMD, EEMD, VMD, and LMD) in bearing fault diagnosis, their inherent limitations under noisy industrial environments warrant critical scrutiny. Empirical decomposition methods (EMD/EEMD) exhibit pronounced sensitivity to noise interference, inducing modal aliasing that obscures fault-related frequency components and precipitates decomposition distortion. While VMD theoretically circumvents mode mixing via variational constraints, its efficacy hinges critically on preset parameters (decomposition level K and penalty factor α), which lack adaptability to signal heterogeneity. Similarly, entropy-based features (e.g., MPE and WPE) suffer from instability under strong noise and high computational overhead in multiscale implementations, limiting their robustness for weak fault detection. These constraints collectively impede reliable feature extraction, necessitating integrated approaches to enhance noise immunity and parameter autonomy. Time-frequency analysis methods (EMD/EEMD) are noise-sensitive and prone to mode mixing and decomposition distortion. Although VMD offers improvements, its requirement for preset decomposition levels remains a limitation. Information entropy methods are sensitive to signal length and noise, incur high computational costs in multiscale analysis, and suffer from feature instability under noise. Traditional classifiers like SVM exhibit performance highly dependent on penalty factors and kernel functions, making parameter tuning difficult and leading to overfitting or reduced accuracy. While scholars globally have achieved significant results in wind turbine bearing fault diagnosis, challenges persist, including signal noise interference and difficulties in acquiring high-quality operational data. Consequently, integrating multiple research methodologies is essential to enhance diagnostic precision and reliability, ensuring the stable and efficient operation of wind turbines. While deep learning architectures (such as convolutional neural networks (CNNs) and transformers) excel in automated feature representation, they confront persistent challenges in industrial fault diagnosis applications. These challenges fundamentally arise from a critical paradox: their substantial dependence on large-scale labeled datasets conflicts directly with the inherent scarcity of actual fault samples. This limitation is exacerbated by restricted cross-domain generalization capability, manifesting as significant performance deterioration under varying operational conditions and further compounded by prohibitive computational costs that impede real-time deployment on resource-constrained edge devices. Collectively, these inherent constraints necessitate the advancement of hybrid methodologies that strategically incorporate prior knowledge of signal processing to achieve robust feature extraction while maintaining computational tractability. In contrast, the approach integrating feature extraction with optimized support vector machines (SVMs) presented in this study offers distinct advantages through its structural simplicity, training efficiency, effectiveness with limited samples, and deployment practicality. These characteristics render it particularly well-suited for scenarios characterized by data scarcity or demanding real-time processing requirements. Consequently, this research focuses on enhancing the robustness and diagnostic accuracy of traditional modeling paradigms, ultimately seeking to deliver an efficient and pragmatically viable solution for engineering applications operating under low-resource constraints.

This study proposes an integrated framework for collaborative bearing fault diagnosis across multiple datasets, combining VMD, refined composite multiscale weighted permutation entropy (RCMWPE) feature extraction, and whale optimization algorithm-optimized support vector machine (WOA-SVM). The core contributions are summarized as follows: Firstly, an innovative integration of VMD signal decomposition, RCMWPE feature extraction, and WOA-SVM classifier establishes synergistic advantages leveraging VMD’s nonstationary signal decoupling capability, RCMWPE’s high robustness feature representation, and WOA-SVM’s parameter optimization effectiveness. Secondly, generalization capability is validated through PRONOSTIA (four class faults) and CWRU (ten class faults) benchmark datasets, confirming cross-dataset scenario adaptability. Thirdly, under complex operating conditions exemplified by the CWRU ten-class task, the system demonstrates diagnostic robustness for nonlinear vibration signals and multimode faults. Finally, experimental results indicate that WOA-SVM achieves 96.5% and 99.67% accuracy rates on PRONOSTIA and CWRU datasets, respectively, significantly outperforming conventional SVM, genetic algorithm-optimized SVM, and particle swarm optimization-optimized SVM baseline models.

## 2. Theoretical Basis and Methodology

This study systematically elaborates the theoretical framework, specifically encompassing the following core methodologies: the application mechanism of VMD in decoupling nonstationary signals; the feature characterization principles of RCMWPE, as shown in Note S1; the high-dimensional feature reduction technique employing t-SNE, as shown in Note S2; the global optimum search mechanism of the WOA; and the decision theoretical foundation of the SVM classifier.

### 2.1. Variational Mode Decomposition

VMD, introduced by Dragomiretskiy and Zosso in 2014 [39], constitutes a non-recursive adaptive signal processing technique grounded in a variational optimization framework; distinct from EMD and its derivatives (e.g., ensemble EMD and complete ensemble EMD), VMD formulates signal decomposition as a constrained variational optimization problem whose essence lies in solving for a set of intrinsic mode functions (IMFs) exhibiting spectral compactness properties; this approach fundamentally circumvents mode aliasing phenomena while guaranteeing exact signal reconstruction through linear superposition of all IMFs, demonstrating significant theoretical rigor and superior robustness against noise compared to conventional recursive decomposition algorithms; its mathematical formulation minimizes the following constrained variational problem:

(1) $$\left\{\sum_{k=1}^{K}\|\begin{array}{c} {𝜕}_{t}\left[\left(\delta \left(t\right)+\frac{j}{\pi t}\right)\times u_{k}\left(t\right)\right]e^{-j\omega_{k}t} \end{array}{\|}_{2}^{2}\right\}+\alpha \sum_{k=1}^{K}\|\begin{array}{c} u_{k}\left(t\right) \end{array}{\|}_{2}^{2}+\langle \lambda \left(t\right),f\left(t\right)-\sum_{k=1}^{K}u_{k}\left(t\right)\rangle$$

In this formulation, *u_k_*(*t*) denotes the *k*-th IMF, *ω_k_* represents the center frequency of the *k*-th IMF, *k* is the preset number of IMFs (modal count), *δ_t_* is the Dirac delta function, × signifies the convolution operation, *j* is the imaginary unit, *e*^−*jωk t*^ serves as the exponential modulation term that shifts each IMF’s spectrum to the baseband, *α* is the quadratic penalty factor balancing reconstruction fidelity and modal bandwidth control, and *λ*(*t*) is the Lagrangian multiplier enforcing the strict reconstruction constraint: $f(t)=\sum_{k=1}^{k}u_{k}(k)$.

The optimization problem is solved iteratively via the alternating direction method of multipliers (ADMM), with specific steps including the following:

1. Initialization: Randomly initialize all IMFs $u_{k}^{1}$ center frequencies $\omega_{k}^{1}$, and Lagrange multipliers $\lambda^{1}$;
2. Iterative Update: Over n iterations, alternately update $u_{k}$ , $\omega_{k}$, and $\lambda^{1}$. Update $u_{k}$: In the frequency domain, the update formula for the *k*-th IMF is

(2) $${\hat{u}}_{k}^{n+1}\left(\omega \right)=\frac{\hat{f}\left(\omega \right)-\sum_{i\neq k}{\hat{u}}_{i}\left(\omega \right)+\frac{\hat{\lambda }\left(\omega \right)}{2}}{1+2\alpha {\left(\omega -\omega_{k}\right)}^{2}}$$

Update $\omega_{k}$: The update formula for the center frequency of the k-th IMF is

(3) $$\omega_{k}^{n+1}=\frac{\int_{0}^{\infty }\omega {\left|{\hat{u}}_{k}^{n+1}\left(\omega \right)\right|}^{2}d\omega }{\int_{0}^{\infty }{\left|{\hat{u}}_{k}^{n+1}\left(\omega \right)\right|}^{2}d\omega }$$

Update λ: The update formula for the Lagrange multiplier is

(4) $$\lambda^{n+1}\left(\omega \right)=\lambda^{n}\left(\omega \right)+\tau \left(\hat{f}\left(\omega \right)-\sum_{k=1}^{K}{\hat{u}}_{k}^{n+1}\left(\omega \right)\right)$$

VMD demonstrates fundamental advantages over EMD and its variants: Its non-recursive architecture eliminates recursive error propagation through parallel mode solving, addressing the cumulative error limitation of EMD-type algorithms. The variational optimization framework combined with ADMM ensures effective mode separation, maintaining compact frequency bands in each IMF. Unlike EMD’s empirical approach, VMD’s rigorous mathematical foundation provides provable decomposition and reproducible results. Furthermore, its parameterized design (e.g., mode count K and penalty factor α) enables adaptive optimization of decomposition structures based on signal characteristics, significantly enhancing the algorithm’s adaptability.

### 2.2. Whale Optimization Algorithm (WOA)

The WOA, proposed by Mirjalili and Lewis in 2016 [40], is a metaheuristic optimization algorithm that mimics the bubble-net feeding behavior of humpback whales. This algorithm demonstrates remarkable advantages in global search capability and convergence speed, making it suitable for solving various optimization problems.

#### 2.2.1. Encircling Prey

Humpback whales can identify and encircle their prey’s position. In WOA, the current best solution is assumed to be the prey’s location, and other whale individuals will move towards it. The mathematical model is as follows:

(5) $$\vec{D}=\left|\vec{C}\cdot {\vec{X}}^{*}\left(t\right)-\vec{X}\left(t\right)\right|$$

(6) $$\vec{X}\left(t+1\right)={\vec{X}}^{*}\left(t\right)-\vec{A}\cdot \vec{D}$$

where *t* represents the current iteration count. $\vec{X}(t)$ denotes the position vector of the current whale individual. ${\vec{X}}^{*}(t)$ indicates the position vector of the current optimal whale individual. $\vec{A}$ and $\vec{C}$ are coefficient vectors calculated as

(7) $$\vec{A}=2\vec{a}\cdot \vec{r}-\vec{a}$$

(8) $$\vec{C}=2\cdot \vec{r}$$

where the vector $\vec{a}$ linearly decreases from 2 to 0 over iterations; $\vec{a}$ is a random vector with components in [0, 1].

Bubble-net attacking is a distinctive foraging behavior unique to humpback whales, comprising two coordinated strategies: encircling contraction and spiral updating.

1. The encircling contraction mechanism is implemented by adjusting the value of vector $\vec{a}$. When |A| < 1, whale individuals move toward the current best solution, simulating the prey-encircling contraction;
2. Spiral position updating mimics whales’ spiral ascent while releasing bubble nets to drive prey toward the water surface. The mathematical model is formulated as

(9) $$\vec{X}\left(t+1\right)={\vec{D}}^{\prime }\cdot e^{bl}\cdot \cos\left(2\pi l\right)+{\vec{X}}^{*}\left(t\right)$$

where $\vec{D}=\left|{\vec{X}}^{*}(t)-\vec{X}(t)\right|$ represents the distance between the whale individual and the optimal solution, b is a constant defining the spiral shape, and l is a random number uniformly distributed in [−1, 1]. During foraging, whales select either the encircling contraction mechanism or spiral position update with 50% probability each. The complete mathematical model is formulated as

(10) $$\vec{X}(t+1)=\left\{\begin{array}{cc} {\vec{X}}^{*}(t)-\vec{A}\cdot \vec{D} & \\ {\vec{D}}^{\prime }\cdot e^{bl}\cdot \cos(2\pi l)+{\vec{X}}^{*}(t) & \end{array}\begin{array}{c} \mathrm{if}\;p<0.5\\ \mathrm{if}\;p\geq 0.5 \end{array}\right.$$

where *p* is a random number uniformly distributed in [0, 1].

#### 2.2.2. Prey Search

When |A| ≥ 1, WOA performs global search by having whale individuals randomly select another whale as a reference and move towards it, simulating the exploratory behavior of humpback whales. The mathematical model is expressed as

(11) $$\vec{D}=\left|\vec{C}\cdot {\vec{X}}_{\mathrm{rand}}-\vec{X}\left(t\right)\right|$$

(12) $$\vec{X}\left(t+1\right)={\vec{X}}_{\mathrm{rand}}-\vec{A}\cdot \vec{D}$$

where $\vec{X}(t)$_rand_ is a position vector randomly selected from the current whale population.

By combining these three mechanisms, WOA achieves a balance between global exploration and local exploitation, demonstrating high efficiency and accuracy when solving complex optimization problems.

### 2.3. Support Vector Machine (SVM)

The SVM is a machine learning method based on statistical learning theory, widely applied to classification and regression tasks [41]. The core concept of SVM involves identifying an optimal hyperplane that maximizes the classification margin between different classes of data points, thereby enabling effective data classification.

#### 2.3.1. Linear SVM for Linearly Separable Data

For linearly separable datasets, SVM aims to find an optimal hyperplane defined by w ⋅ x + b = 0 that maximizes the sum of distances from sample points of both classes to the hyperplane. This distance is termed the classification margin. Mathematically, the problem can be formulated as the following optimization problem:

(13) $$\underset{w,b,\xi }{\min}\frac{1}{2}∥w{∥}^{2}+C\sum_{i=1}^{N}\xi_{i}$$

(14) $$s.t.y_{i}\left(w\cdot x_{i}+b\right)\geq 1,\;i=1,\ldots ,N$$

where *x_i_* represents the training sample, *y_i_* ∈ {−1, +1} denotes the corresponding class label, w is the normal vector to the hyperplane, and b is the bias term.

#### 2.3.2. Nonlinear SVM and Kernel Functions

In practical applications where many datasets are not linearly separable, SVM addresses this challenge by introducing soft margin techniques and kernel functions. The soft margin approach permits limited margin constraint violations through the use of slack variables *ξ_i_* ≥ 0 and a penalty parameter C > 0 to accommodate outliers and noise, leading to the modified optimization problem:

(15) $$\underset{w,b,\xi }{\min}\frac{1}{2}∥w{∥}^{2}+C\sum_{i=1}^{N}\xi_{i}$$

(16) $$\underset{w,b}{\min}\frac{1}{2}∥w{∥}^{2}$$

(17) $$s.t.y_{i}\left(w\cdot x_{i}+b\right)\geq 1-\xi_{i},\;\xi_{i}\geq 0,\;i=1,\ldots ,N$$

Kernel Functions: When data are linearly inseparable in the original space, SVM employs a nonlinear mapping *ϕ*(*x*) to project the data into a higher-dimensional feature space, where linear separation becomes possible. The kernel function $K(x_{i},x_{j})=\phi (x_{i})\cdot \phi (x_{j})$ computes inner products in this high-dimensional space directly within the original space, bypassing explicit feature mapping and significantly reducing computational complexity. Common kernel functions include

Linear Kernel Function: $K(x_{i},x_{j})=x_{i}\cdot x_{j}$
Polynomial Kernel Function: $K(x_{i},x_{j})={\left(\gamma x_{i}\cdot x_{j}+r\right)}^{d}$
Radial Basis Function (RBF) Kernel: $K(x_{i},x_{j})=\exp(-\gamma {\left\|x_{i}-x_{j}\right\|}^{2})$ denotes the kernel function.

This study employs the radial basis function (RBF) kernel for SVM due to its capability in handling nonlinear relationships and strong generalization performance. The effectiveness of the RBF kernel is primarily governed by two key parameters: C (penalty parameter) and *γ* (kernel coefficient). Applying optimization algorithms to fine-tune these parameters can significantly enhance the classification performance of SVM.

## 3. Experimental Setup and Data Analysis

**Figure 1:**
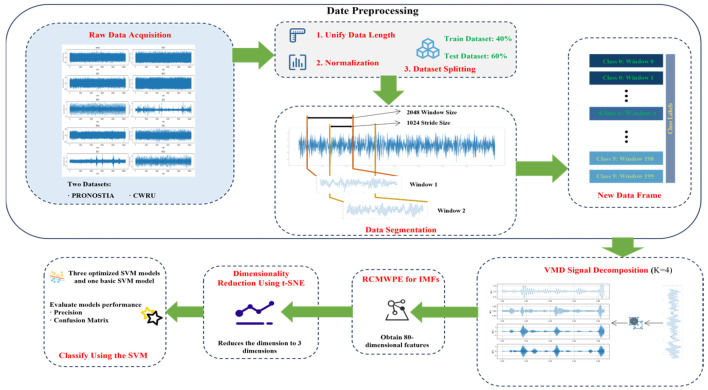
Schematic Diagram of the Overall Bearing Fault Diagnosis Methodology. Raw vibration signals, sourced from the PRONOSTIA and CWRU datasets, undergo preprocessing including normalization, segmentation, and annotation. Subsequently, VMD is employed for signal decomposition, followed by high-dimensional feature extraction using RCMWPE. The extracted features are then visualized via dimension reduction with t-SNE. Finally, classification is performed using four distinct SVM models, encompassing three optimized variants.

This section systematically elaborates the core elements of the PRONOSTIA and CWRU bearing fault datasets employed in this study, encompassing each dataset’s experimental platform configurations, fault mode typologies, signal acquisition specifications, and preprocessing workflows. It further details the operational procedures for VMD signal decomposition, RCMWPE feature extraction, and t-SNE dimensionality reduction implemented on the respective datasets, as shown in Figure 1.

## 4. PRONOSTIA Dataset

### 4.1. Dataset Description

**Figure 2:**
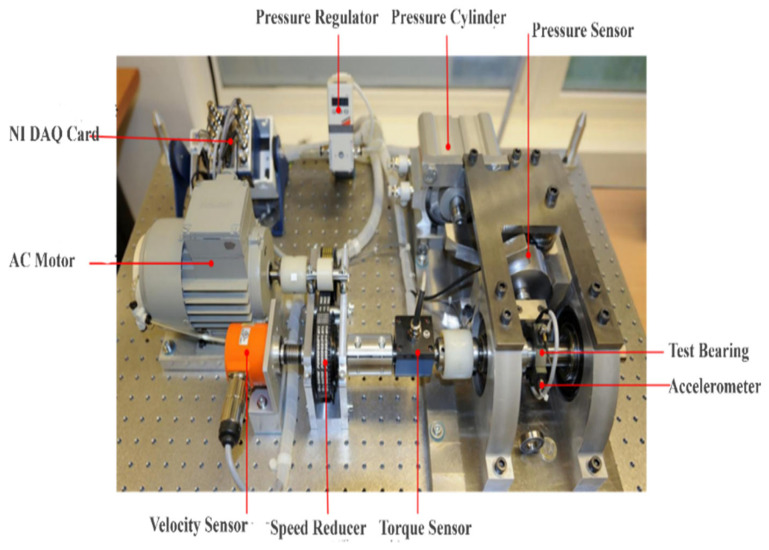
This platform comprises a rotational module, a regulation module, and a measurement module. Its primary objective is to produce verifiable data spanning the full lifecycle of bearings, from healthy states to complete failure under various impact conditions.

The PRONOSTIA dataset is generated by a dedicated bearing fault experimental platform developed by the AS2M Department of FEMTO ST Institute, France [42]. As illustrated in Figure 2, this platform comprises a rotational module, a regulation module, and a measurement module. Its primary objective is to produce verifiable data spanning the full lifecycle of bearings, from healthy states to complete failure under various impact conditions.

**Figure 3:**
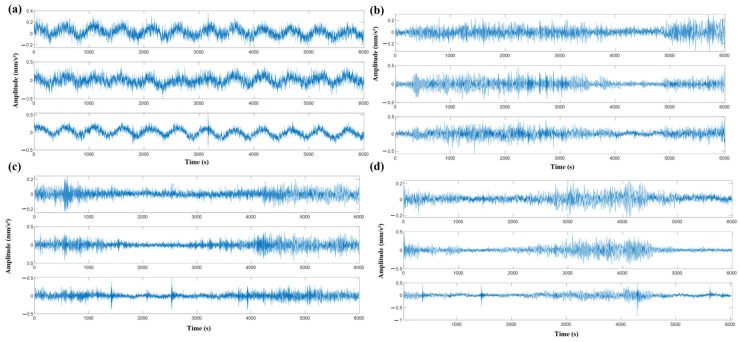
Time-domain vibration waveforms along triaxial (X, Y, Z) orientations under varying operational conditions. Subplots (**a**–**d**) present raw vibration signals acquired in three orthogonal directions for four representative states (labeled 1–4). Each state corresponds to distinct fault types or health conditions, exhibiting discernible signal feature discrepancies that substantiate subsequent feature extraction and classification.

The PRONOSTIA platform exhibits distinctive features differing from onshore wind turbine bearing data, encompassing comprehensive defect types including balls, rings, and cages. Vibration signals are acquired through orthogonally distributed (90° angular configuration) biaxial triaxial accelerometers radially mounted on the bearing outer race for acceleration measurement. A sampling frequency of 25.6 kHz is implemented with an intermittent sampling protocol (1 s acquisition within 10 s cycles), yielding 2560 data points per sampling instance, as shown in Figure 3. Three operating conditions are configured: 1800 rpm/4000 N, 1650 rpm/4200 N, and 1500 rpm/5000 N. As delineated in Table 1, the dataset comprises four operational modes: healthy state, inner ring defects, rolling element defects, and outer ring defects.

### 4.2. Data Preprocessing and Feature Extraction

During the preprocessing phase, preliminary analysis was conducted on the acquired raw data to eliminate invalid entries and samples with missing values. To augment the usable data volume, an overlapping sampling strategy was implemented on initial bearing vibration signals, and normalization was applied to simulated sample datasets. For distinct operational states, 180 vibration signal samples were collected along each of the X, Y, and Z axes, resulting in 720 sample sets across four operational states, with each sample containing 4096 consecutive data points. Subsequently, to extract deep features from raw vibration signals reflecting diverse bearing health conditions, VMD was separately applied to triaxial signals. During decomposition, the modal component count K was optimized via the central frequency observation method to ensure effective signal decomposition. For instance, when decomposing simulated vibration signals under severely worn conditions, varying K values induced corresponding shifts in central frequencies (specific values detailed in Table 2).

**Figure 4:**
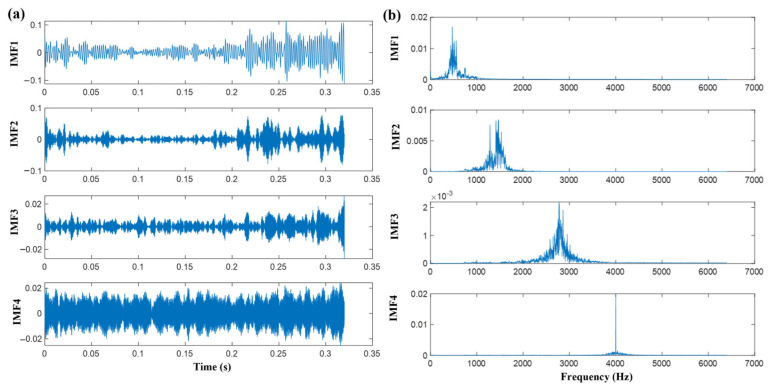
(**a**) Temporal patterns of four IMFs extracted via VMD (K = 4), demonstrating multiscale decomposition characteristics of the original vibration signal. (**b**) Fourier spectra corresponding to each IMF under accelerated wear state. Spectral energy concentrations indicate band-limited fault signatures essential for diagnostic feature extraction.

To determine the appropriate modal number (K) for VMD, this study analyzed the influence of different K values on central frequencies. Analysis results demonstrate that at K = 5, the central frequencies of the second intrinsic mode function (IMF2) and third intrinsic mode function (IMF3) were excessively proximate, indicating over decomposition. Consequently, K = 4 was ultimately selected for VMD decomposition. This algorithm exhibits inherent noise robustness through its bandwidth constraint mechanism, facilitating noise interference suppression during signal reconstruction. To preserve critical signal details while effectively suppressing noise, a penalty factor was introduced during decomposition. Applying K = 4 to raw signals yielded time domain waveforms and corresponding frequency spectra for each IMF, as shown in Figure 4. No modal mixing or under decomposition was observed, confirming successful extraction of essential feature information from original vibration signals. To evaluate the performance advantages of VMD in vibration signal processing, this study conducted comparative analysis with EMD, EEMD, and CEEMD using identical datasets. Decomposition effectiveness was quantitatively compared by calculating cross-correlation coefficients between the first four IMFs obtained by each algorithm and the original signals, with detailed comparative cross-correlation values presented in Table 3.

**Figure 5:**
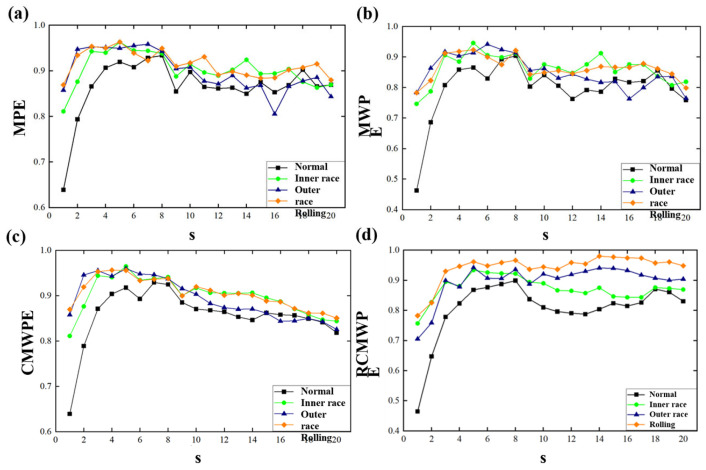
(**a**–**d**) Comparative entropy trajectories of MPE, MWPE, CMWPE, and RCMWPE across multiscale factors. The plots demonstrate processing outcomes for pitch bearing vibration signals under distinct wear degradation states, with identical parameterization (embedding dimension m = 5, maximum scale factor s = 20, time delay *τ* = 1, and sequence length *N* = 2048).

Post decomposition analysis revealed that correlation coefficients between VMD derived modal components predominantly ranged from 0.5 to 1, confirming that intrinsic mode functions IMF1 through IMF4 effectively captured core characteristics of the original signal with significant information representation capability. In contrast, EMD modal coefficients exhibited higher dispersion, particularly with IMF4 correlation coefficients below 0.1, indicating severe feature information deficiency. This comparative outcome underscores VMD’s substantial advantage in preserving modal validity, establishing the technical foundation for feature reconstruction. Consequently, VMD was employed for bearing vibration signal processing in this study. Subsequently, RCMWPE feature extraction was applied to VMD reconstructed components. Based on feature parameter optimization results under Gaussian white noise conditions, parameters were configured with embedding dimension m = 5, maximum scale factor s = 20, delay time τ = 1, and sequence length N = 2048. To validate RCMWPE superiority, performance comparisons were conducted against MPE, MWPE, and CMWPE under identical parameters, with comparative results depicted in Figure 5.

**Figure 6:**
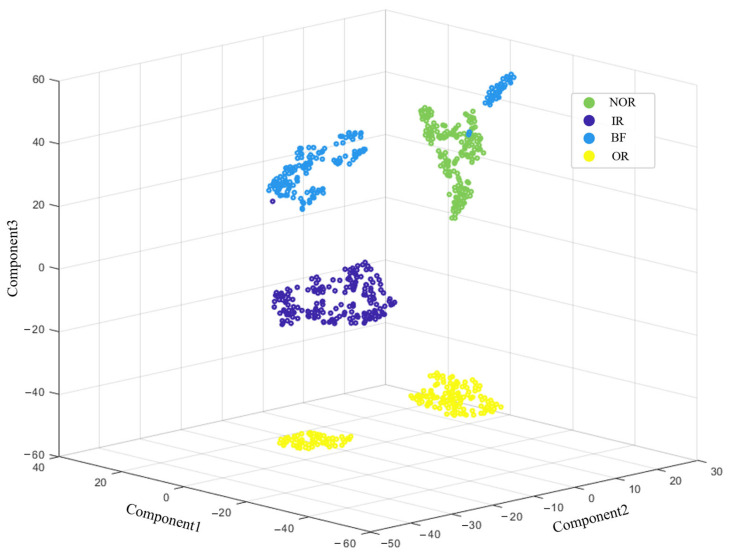
Three-dimensional t-SNE visualization of bearing fault signatures based on RCMWPE features (PRONOSTIA dataset), with color-coded representations indicating distinct failure modes: normal (NOR), inner raceway fault (IR), outer raceway fault (OR), and ball fault (BF).

As illustrated in Figure 5, entropy curves of MPE, MWPE, CMWPE, and RCMWPE exhibit an initial ascent followed by descent as scale factors increase. Notably, MPE and MWPE demonstrate significant fluctuations across global scale ranges with insufficient discriminative capacity for different wear states of pitch bearings. Conversely, CMWPE and RCMWPE entropy curves display superior smoothness and stability. When scale factors exceed 4, RCMWPE manifests pronounced feature separability across distinct wear states, indicating its extracted feature vectors effectively characterize pitch bearing degradation levels with robust generalization capabilities. Although the 80 dimensional feature vectors extracted via RCMWPE contain rich state information, their high dimensionality impedes three dimensional visualization. Therefore, t-SNE nonlinear dimensionality reduction was employed to map high-dimensional features into low-dimensional space, leveraging its topological preservation capability to intuitively reveal intrinsic data structures and pattern distributions, with dimensionality reduction results presented in Figure 6.

As depicted in Figure 6, the data samples of distinct states exhibit significant spatial distribution patterns: samples of identical states form compact cluster structures, while feature sets of different states demonstrate clear interclass separation with no category overlap observed. This distribution characteristic confirms the superior representational capacity of the extracted high-dimensional features, enabling effective identification of pitch bearing wear severity variations.

### 4.3. CWRU Dataset

**Figure 7:**
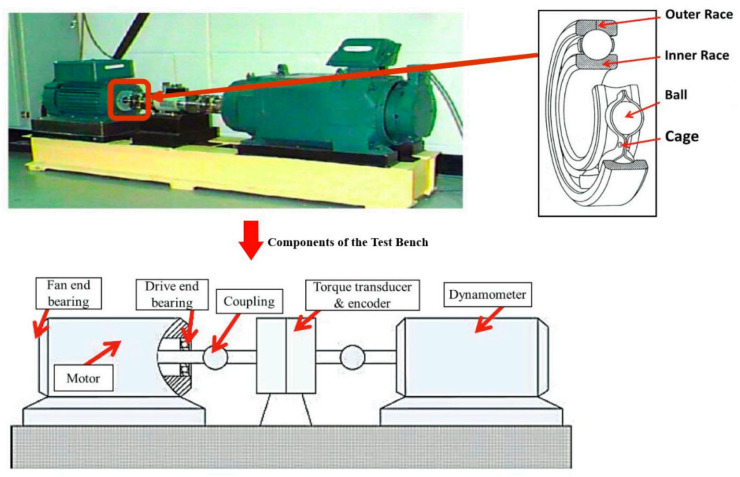
CWRU Motor experimental [43].

This study employs the Case Western Reserve University (CWRU) bearing dataset [10] as an independent benchmark source for methodology validation. As illustrated in Figure 7, the dataset was acquired via a motor experimental platform, with vibration signals captured using triaxial accelerometers mounted directly above the drive-end (DE) and fan-end (FE) bearing housings.

#### 4.3.1. Dataset Description

This study employs the CWRU dataset for evaluation the proposed methodology. Figure 7 illustrates the CWRU motor experimental apparatus utilized for investigating ball bearing defects. Vibration data were acquired through three accelerometers mounted at the 12 o’clock position on the DE and FE housing. The DE and FE assemblies incorporated SKF 6205-2RS JEM and 6203-2RS JEM (SKF Group) deep groove ball bearings, respectively. Artificially induced faults ranging in diameter from 0.007 to 0.021 inches were introduced into the bearings using electrical discharge machining. Vibration signals were sampled at 48 kHz, corresponding to motor rotational speeds varying between 1720 rpm and 1797 rpm. Three distinct motor loading conditions were implemented: no load (L0, 0% of nominal load), half load (L1, 50% of nominal load), and full load (L2, 100% of nominal load).

**Figure 8:**
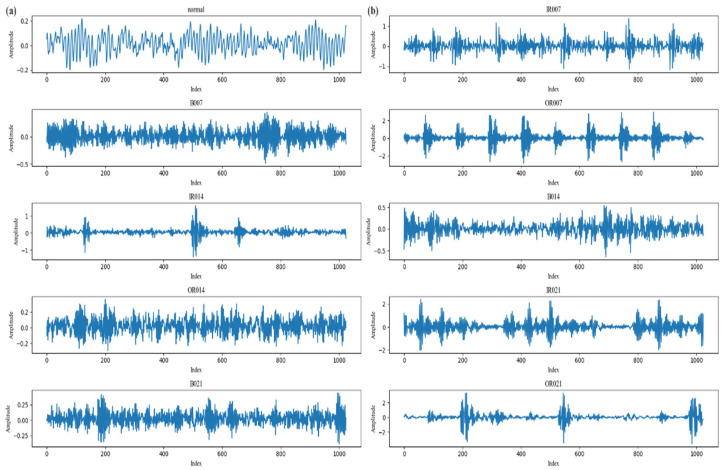
(**a**,**b**) Characteristic 1024-sample vibration signatures for ten distinct conditions (including healthy state and diverse fault typologies) extracted from the CWRU dataset. Discriminative variations in temporal amplitude profiles and waveform structures reveal fault-specific excitation patterns in vibrational responses.

Consequently, this investigation utilization the Case Western Reserve University (CWRE) bearing dataset, which comprises four principal data categories: normal baseline data, DE fault data sampled at 12 kHz, DE fault data sampled at 48 kHz, and FE fault data sampled at 12 kHz. the dataset incorporates triaxial acceleration signals from the DE, FE, and base (BA), accompanied by corresponding rotational speed measurements, with DE signals available at both 12 kHz and 48 kHz sampling frequencies, while FE signals are exclusively provided at 12 kHz. For the current experimental protocol, data extraction adhered to the following specifications: exclusive selection of 0 horsepower (0 HP) operational condition data; utilization of baseline data for normal conditions; and restriction to 12 kHz-sampled DE signals for fault conditions. The selected fault types comprise three defect modalities, specifically inner race (IR), ball, and outer race (OR) faults, each exhibiting three discrete fault severity levels corresponding to diameters of 0.007, 0.014, and 0.012 inches. Integration of the normal baseline state with the nine resultant fault conditions, formed through the combination of three defect modalities and three severity levels, generated a comprehensive ten-class classification dataset, as shown in Figure 8 and Table 4.

#### 4.3.2. Data Preprocessing and Feature Extraction

**Figure 9:**
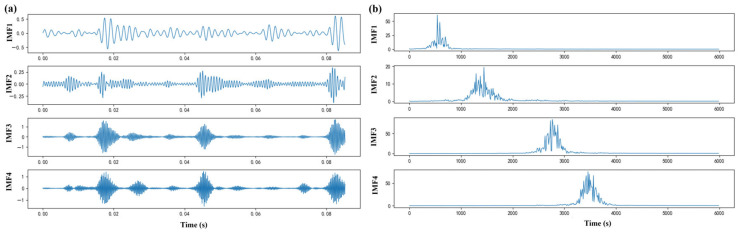
(**a**) Temporal waveforms of intrinsic mode functions (IMFs) derived from variational mode decomposition (VMD) of vibration signals under OR021 condition (outer race fault, 0.021-inch damage). The decomposition with parameter *K* = 4 reveals multiscale temporal characteristics embedded in the raw signal. (**b**) Spectral distributions of corresponding VMD-generated IMFs. Dominant frequency components within each IMF elucidate band-limited fault signatures essential for resonance frequency identification.

To quantitatively evaluate the feature preservation capabilities of different signal decomposition methods, this study computed Pearson correlation coefficients between the original vibration signal and the first four IMFs extracted through EMD, ensemble EMD (EEMD), complete EEMD with adaptive noise (CEEMDAN), and VMD. Analytical results reveal that while IMF1 components from EMD, EEMD, and CEEMDAN exhibit high correlation with the original signal, their subsequent IMF components demonstrate precipitous correlation coefficient declines, indicative of energy concentration and mode mixing phenomena. In contrast, the IMF3 and IMF4 components derived from VMD decomposition maintain significant correlations (coefficients: 0.5954 and 0.7145, respectively), evidencing effective isolation of mid-to-low frequency components without spurious mode interference. Collectively, these findings demonstrate VMD’s superior performance in signal representation fidelity and modal decoupling capability, as shown in Figure 9 and Table 5.

This study extends the established methodological framework to higher-complexity fault diagnosis scenarios through the development of a ten-category diagnostic model. Whereas prior research was constrained to quadripartite classification tasks, the current work utilizes the CWRU bearing dataset under 0-hosepower (0 HP) operating conditions. The diagnostic scope now encompasses ten distinct fault states across three defect modalities: inner race (IR), ball, and outer race (OR) faults. Each modality manifests three damage severity levels (0.007, 0.014, and 0.021 inches in diameter). This expansion substantially elevates the model’s representation capability for complex fault patterns and extends its practical applicability boundaries

This investigation adheres to the original methodological framework: initially employing VMD for vibration signal preprocessing and decomposing signals in four IMFs; subsequently utilizing RCMWPE to extract features from IMF components, thereby constructing a high-dimensional feature space; and further implementing feature visualization through t-distributed stochastic neighbor embedding (t-SNE) nonlinear dimensionality reduction. As illustrated in Figure 9, the resulting 2D projection distinctly reveals spatially discernible cluster distributions across ten fault categories, empirically verifying the enhanced separability capacity of the feature extraction scheme for complex fault patterns.

### 4.4. Impact of the VMD Penalty Factor α on Feature Extraction Performance

To quantify the impact of VMD’s penalty factor α on diagnostic efficacy, parameter sensitivity analysis was conducted on the noise-free CWRU dataset. As depicted in Table 6, classification accuracy varies with α values (100–2500): lower α induces spectral overlap across IMFs, impairing feature discriminability, while excessive α causes modal information loss. Optimal performance (WOA-SVM accuracy > 99.2%) occurs within α∈ [1500, 2500], justifying our selection of α = 2000. This confirms VMD’s critical role in balancing mode separation and feature preservation.

## 5. Fault Diagnosis Model Development and Result Analysis

This section systematically elaborates upon the construction process of a bearing fault diagnosis model integrating variational mode decomposition–refined composite multiscale weighted permutation entropy (VMD-RCMWPE) feature extraction and WOA-SVM classification while conducting a multidimensional comparative analysis and discussion of experimental results obtained from the PRONOSTIA platform and the CWRU dataset.

### 5.1. Based on WOA-SVM Fault Diagnosis Model

The selection of SVMs as the baseline classifier in this work is primarily justified by their intrinsic model characteristics aligning profoundly with the research requirements and methodological rigor. SVMs demonstrate superior generalization capabilities for small sample sizes, high-dimensional data, and nonlinear distributions within the framework of statistical learning theory, a property exhibiting high compatibility with this study’s data structure. Their established effectiveness and robustness in practical engineering applications such as mechanical fault diagnosis are well-supported by empirical evidence. Compared to complex models like deep neural networks, SVMs offer significantly higher architectural determinism and training efficiency. This facilitates effective synergistic integration with the proposed variational mode decomposition VMD-RCMWPE feature extraction method, thereby mitigating potential confounding effects of classifier complexity on feature effectiveness evaluation. Crucially, given this research’s core focus on enhancing model robustness under strong noise conditions and validating the efficacy of optimization algorithms like the WOA for parameter tuning, employing the theoretically mature and parametrically transparent SVM as a benchmark enables strict variable control. This controlled approach permits clear quantification of the independent contributions from feature engineering methodologies and optimization strategies to performance enhancement, ensuring an objective assessment of methodological innovation. The model construction commences with the acquisition of raw vibration signals from the PRONOSTIA and CWRU bearing datasets, followed by sliding window segmentation and normalization preprocessing to eliminate dimensional influences; subsequent VMD processing, with the modal number uniformly set to K = 4 according to Section 4.2 analytical conclusions, decouples the original signals into a series of IMFs; RCMWPE feature extraction is then applied to each IMF component using parameter configurations including embedding dimension m = 5, maximum scale factor s = 20, time delay τ = 1, and sequence length N = 1024, integrating features from all IMF components to construct a high-dimensional feature vector; t-SNE dimensionality reduction subsequently maps these features into a two-dimensional space for feature validation visualization and classification input simplification; the dimensionality-reduced features are then input into a SVM, where the WOA performs global optimization of the penalty parameter C and radial basis kernel parameter γ using validation set classification accuracy as the fitness function; finally, the optimized SVM model executes fault classification to output diagnostic results.

### 5.2. Experimental Results and Analysis on the PRONOSTIA Dataset

To validate the universality of the proposed methodology in multistate bearing fault diagnosis, a four-class classification experiment was conducted using the PRONOSTIA dataset; comparative performance evaluation was performed between the WOA-SVM and benchmark models, including standard SVM, genetic algorithm-optimized SVM (GA-SVM), and particle swarm optimization-optimized SVM (PSO-SVM), through partitioned training/testing sets; as documented in Table 5, WOA-SVM achieved a classification accuracy of 96.5%, substantially surpassing baseline models (representing a 14.2% improvement over standard SVM, 7.8% over GA-SVM, and 5.3% over PSO-SVM), with its confusion matrix further demonstrating optimal inter-class separability; these findings confirm that WOA significantly enhances the model’s out-of-sample classification generalizability and diagnostic precision by virtue of its efficient exploration of the optimal solution space for the penalty parameter *C* and radial basis kernel parameter *γ*.

**Figure 10:**
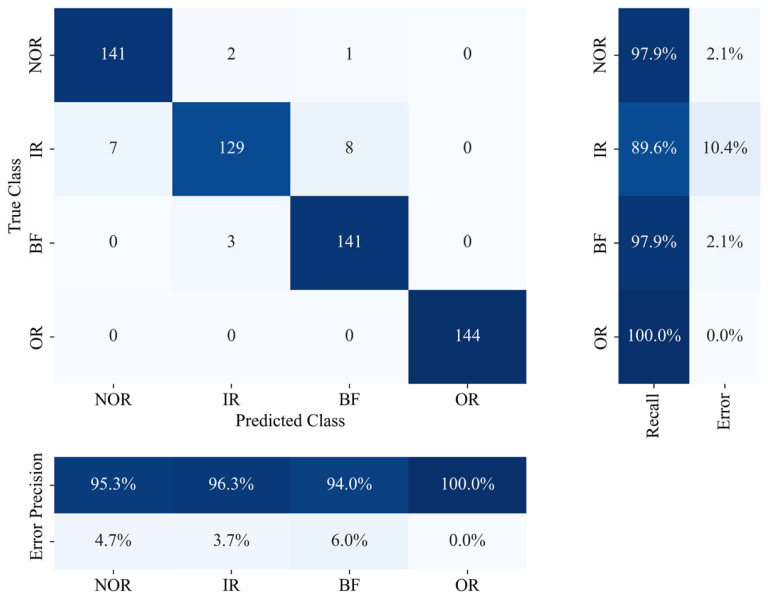
Confusion Matrix for the WOA-SVM Model on the PRONOSTIA Dataset Confusion Matrix for the WOA-SVM Model on the PRONOSTIA Dataset, Normal (NOR), Inner Raceway Fault (IR), Outer Raceway Fault (OR), and Ball Fault (BF).

Figure 10 presents the confusion matrix of the WOA-SVM model on the PRONOSTIA dataset; this visualization quantitatively reveals the model’s discriminative accuracy across four bearing health states through systematic assessment of classifier confusion characteristics among different fault patterns, as evidenced by correctly classified samples represented by diagonal elements and misclassification distributions reflected by off-diagonal elements.

As illustrated in the confusion matrix of Figure 10 and Table 7, the WOA-SVM model achieves recognition accuracy exceeding 94% for both the normal condition and three fault states: inner race (IR), ball, and outer race (OR). Limited misclassifications predominantly occur as inter-class confusion between inner race and ball faults (less than 5%), attributable to their partial feature overlap in the time-frequency domain. Overall, the model demonstrates exceptional inter-class separability with an average classification accuracy of 96.5%, confirming its efficacy in discerning inherent variations among bearing health conditions.

For fault classification tasks, this research implements an integrated framework combining WOA with SVM, designated as WOA-SVM, and systematically evaluates its performance against standard SVM, GA-SVM, and particle swarm optimization-enhanced SVM (PSO-SVM). Quantitative assessment of the decametric classification performance was conducted through confusion matrix analysis, with comparative results delineated in subsequent sections.

### 5.3. Experimental Results and Analysis on the CWRU Dataset

**Figure 11:**
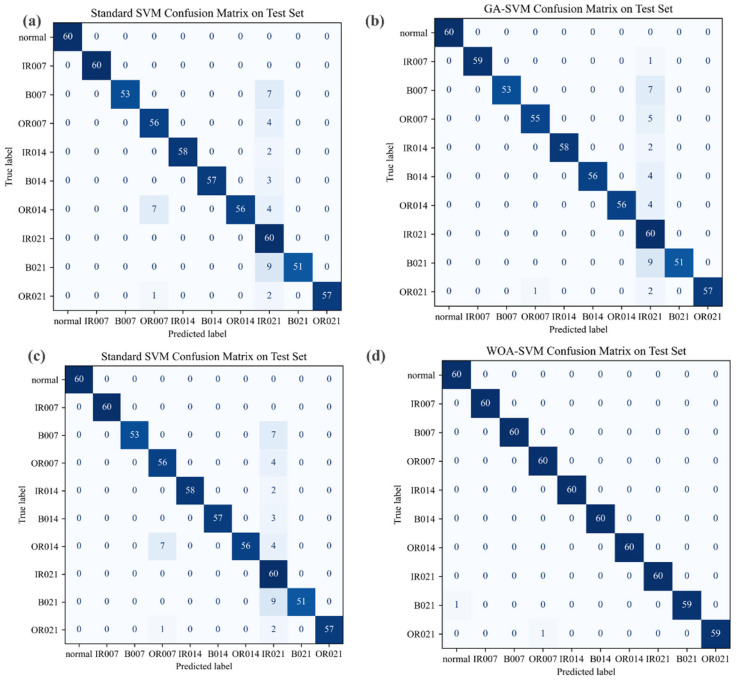
(**a**) Confusion Matrix of the SVM Model on the CWRU Test Set; (**b**) Confusion Matrix of the GA-SVM Model on the CWRU Test Set; (**c**) Confusion Matrix of the PSO-SVM Model on the CWRU Test Set; (**d**) Confusion Matrix of the WOA-SVM Model on the CWRU Test Set.

The diagnostic performance of each classification model on the test set was systematically evaluated through confusion matrix analysis, with principle findings indicating that the WOA-SVM model achieved a test accuracy of 99.67%, demonstrating statistically significant superiority over benchmark methods, including standard SVM, GA-SVM, and PSO-SVM, as shown in Table 8. This optimized classifier exhibits exceptional capability in discriminating subtle fault patterns, maintaining robust classification performance particularly for morphologically similar failure types such as outer race faults at distinct damage locations and minor defects at the 0.007-inch severity level. The successful validation of this decametric classification scenario empirically substantiates the enhanced robustness and engineering applicability of the VMD-based signal processing framework for complex fault diagnosis task, as shown in Figure 11 and Table 8.

## 6. Comprehensive Discussion

Integrating experimental results from both PRONOSTIA and CWRU datasets, the proposed VMD-RCMWPE feature extraction and WOA-SVM classifier integration methodology demonstrates significant advantages in bearing fault diagnosis: cross-dataset validation confirms its strong robustness (96.5% accuracy for four-class classification on PRONOSTIA, 99.67% for ten-class classification on CWRU); VMD effectively resolves nonlinear signal mode aliasing to deliver high-quality intrinsic mode functions; RCMWPE extracts highly discriminative time-frequency features through multiscale complexity quantification; WOA achieves global optimization of SVM penalty parameter *C* and kernel parameter *γ*, yielding a 7.3-percentage-point accuracy improvement over genetic algorithm/particle swarm optimization counterparts; and the methodology’s generalization capability across four-class to ten-class scenarios validates its engineering applicability, thereby establishing reliable technical support for early-stage fault warning in industrial equipment.

### 6.1. Classification Accuracy Under Varied Noise Conditions

To comprehensively validate the noise robustness of the proposed methodology, a ten-class CWRU dataset was employed to construct six experimental noise environments with signal-to-noise ratio (SNR) gradients ranging from −5 dB to 20 dB. Systematic comparisons were conducted on the fault classification performance among (i) standard SVM, (ii) GA-SVM, (iii) PSO-SVM, and (iv) the proposed WOA-SVM. As shown in Table 9, the experimental results demonstrate that under low-SNR conditions (SNR = −5 dB), conventional SVM accuracy significantly decreased to 71.33%, whereas WOA-SVM maintained 77.17% accuracy. When SNR increased to 0 dB, WOA-SVM achieved 95.83% accuracy (compared to 82.00% for conventional SVM). In medium-to-high SNR scenarios (SNR ≥ 5 dB), WOA-SVM consistently sustained classification accuracy exceeding 98% (peaking at 99.67%), outperforming both GA-SVM and PSO-SVM across most noise levels. These findings conclusively establish the superior noise immunity and generalization capability of WOA-SVM, particularly for industrial fault diagnosis applications subject to significant background noise interference.

To evaluate the robustness of the proposed method against data missingness in practical industrial applications, this study constructed datasets with varying missing rates (5%, 10%, 20%, 30%, and 40%) under the missing completely at random (MCAR) mechanism, using the original complete dataset as the baseline. Missing values were imputed through three established methods: forward filling (ffill), linear interpolation (linear), and cubic spline interpolation (spline). All datasets then underwent identical sequential processing involving VMD, RCMWPE feature extraction, t-SNE dimensionality reduction, and WOA-SVM classification. Five independent training and testing trials were performed for each combination of missing rate and imputation method. The mean values and standard deviations of performance metrics including average accuracy (Acc), macro-averaged F1-score (macro-F1), and Cohen’s kappa coefficient (κ) were subsequently calculated and documented in Table 10. This table presents the performance metrics of the WOA-SVM classifier on the test set under various missing rates (5%, 10%, 20%, 30%, and 40%) and imputation methods (forward filling/ffill, linear interpolation/linear, and cubic spline interpolation/spline) within the missing completely at random (MCAR) framework. Reported metrics include average accuracy (Acc), macro-averaged F1-score (macro-F1), and Cohen’s kappa coefficient (κ), accompanied by their standard deviations (±SD). Experimental results demonstrate that all three imputation methods maintained classification performance comparable to the original complete dataset (baseline) at lower missing rates, while cubic spline interpolation exhibited comparatively superior robustness under elevated missing rate conditions.

The table demonstrates strong numerical and trend consistency across all three metrics, where Acc serves as the most intuitive indicator of overall classification correctness. The macro-averaged F1-score (macro-F1), which assigns equal weight to all classes in this multiclassification task, evaluates model balance under potential class distribution imbalances. The near-equivalence between macro-F1 and Acc indicates consistent classification performance across all classes without significant degradation in any specific category. Cohen’s kappa coefficient (κ) quantifies classification agreement with ground-truth labels while accounting for random chance, showing slightly lower absolute values than Acc but identical trends, confirming that performance variations primarily stem from overall classification precision changes rather than class imbalance or random effects.

At missing rates up to 10%, all imputation methods maintained stable high-precision classification, with cubic spline interpolation at 5% missing rate approaching the original dataset’s performance. This may be attributable to imputation-induced signal smoothing that suppresses high-frequency noise components and enhances feature discriminability. Progressive performance degradation occurred with increasing missing rates: forward ffill showed highest sensitivity (94.5% Acc at 40% missing rate), linear interpolation exhibited moderate but significant deterioration, while cubic spline interpolation maintained superior performance across most conditions, preserving 98.0% accuracy even under a 40% missing rate, demonstrating exceptional time-series pattern recovery capabilities.

**Figure 12:**
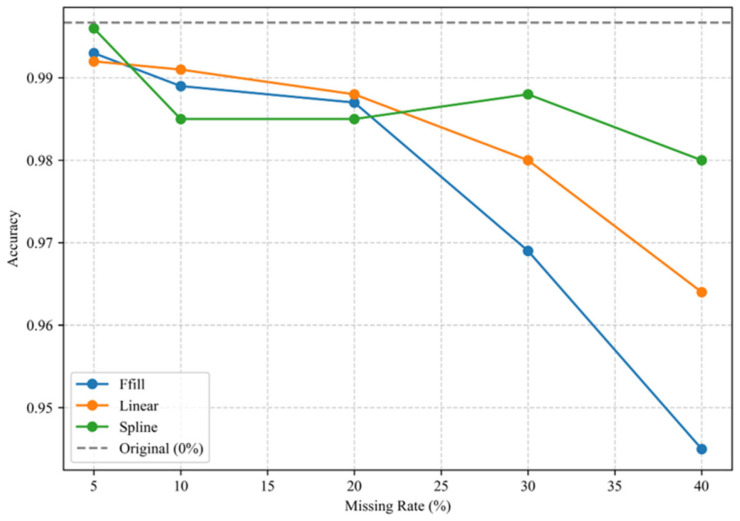
Impact of missing rates and imputation methods on WOA-SVM classification accuracy.

Figure 12 depicts the accuracy trends of three imputation methods across varying missing rates. Under low missing rates (≤10%), all accuracy curves remain proximate to the baseline performance level, whereas significant divergence emerges among methods at elevated missing rates. The cubic spline curve exhibits the most gradual decline, demonstrating its robustness advantage in high-missing-data scenarios. Collectively, the proposed WOA-SVM framework maintains high classification performance despite substantial missing-data perturbations, confirming its applicability in industrial monitoring settings where complete data integrity cannot be guaranteed.

### 6.2. Significance Testing for Performance Divergence

**Figure 13:**
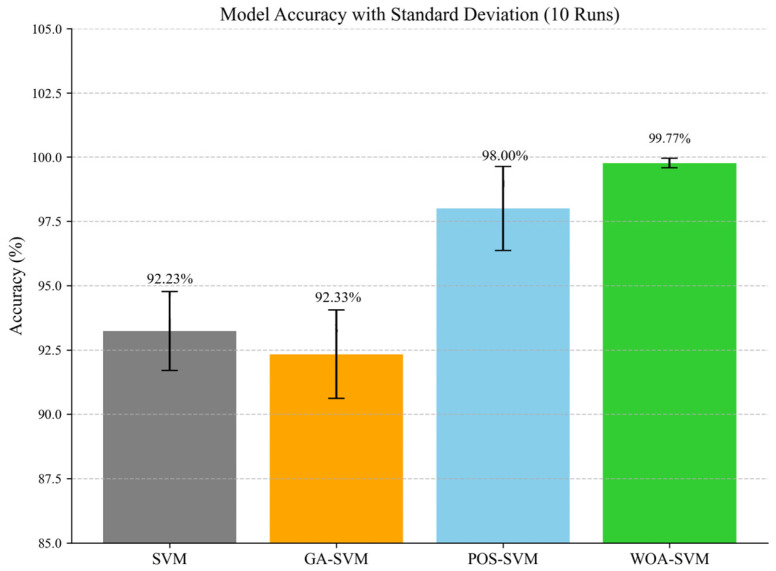
Comparative Performance of SVM Models in Bearing Fault Classification: Mean Accuracy and Standard Deviation over 10 Independent Trials.

To comprehensively evaluate the effectiveness of optimized models for bearing fault classification, this study conducted ten independent repeated experiments under identical data conditions comparing the standard SVM, GA-SVM, PSO-SVM, and the proposed WOA-SVM. The experimental results (mean classification accuracy with standard deviations) are presented in Figure 13.

Experimental results indicate that the proposed WOA-SVM achieved a mean classification accuracy of 99.77% (SD = 0.19%), significantly outperforming all comparative models while demonstrating exceptional stability and robustness. PSO-SVM ranked second with 98.00% accuracy, whereas GA-SVM performed marginally below the standard SVM baseline. As shown in Table 11, To validate the statistical significance of these performance differences, paired *t*-tests were conducted against the reference SVM model at a significance level of α = 0.01. The analysis revealed highly significant differences for both PSO-SVM and WOA-SVM (*p* < 0.01), with WOA-SVM attaining an extreme significance level of *p* < 0.0001—confirming the high statistical reliability of its performance enhancement.

### 6.3. Generalization Capability Assessment

**Figure 14:**
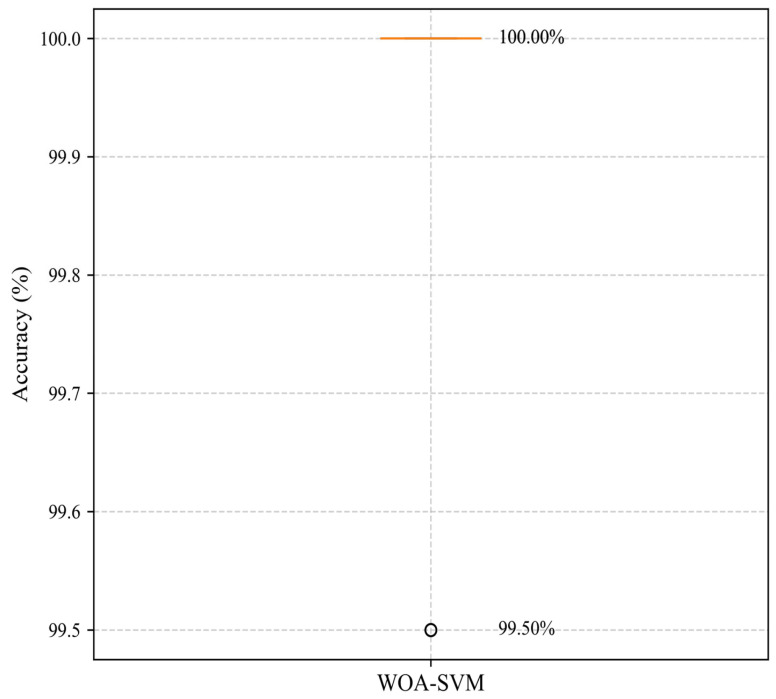
Accuracy distribution of WOA-SVM using 5-fold cross-validation.

To validate the generalization capability of WOA-SVM, we implemented 5-fold cross-validation with the following classification accuracies across folds: 99.50%, 100.00%, 100.00%, 100.00%, and 100.00%. The resultant mean accuracy was 99.90% (SD = 0.20%). These findings demonstrate that the model maintains consistently superior performance across diverse data partitions, effectively mitigating overfitting risks. The high accuracy rates achieved exhibit robust statistical credibility, as shown in Figure 14.

### 6.4. Independent Test Set Performance Evaluation

To address reviewer concerns regarding potential overfitting suggested by high accuracy rates, this study conducted supplementary performance evaluation and generalization validation on an independent test set. The MCAR5_linear scenario involves randomly deleting 5% of sample values from the original dataset under the missing completely at random (MCAR) mechanism, with missing values reconstructed through linear interpolation. This configuration simulates minor random data loss in industrial data acquisition while examining the model’s stable feature extraction and classification capabilities following basic data repair.

The experimental procedure maintained consistent feature extraction through VMD and RCMWPE. Extracted training features were input into the WOA-SVM model, which utilized the macro-averaged F1-score (macro-F1) from 5-fold cross-validation as its fitness function. Optimal penalty parameter *C* and kernel coefficient γ were independently searched under four random seeds (42 to 45). Models were retrained using optimized hyperparameters and evaluated on independent test sets with confusion matrices recorded. Learning curves based on macro-F1 were plotted under the initial random seed, where “CV-Validation” denotes average validation performance via 5-fold cross-validation (*k* = 5). These curves reveal fitting and generalization trends during incremental training sample expansion.

**Figure 15:**
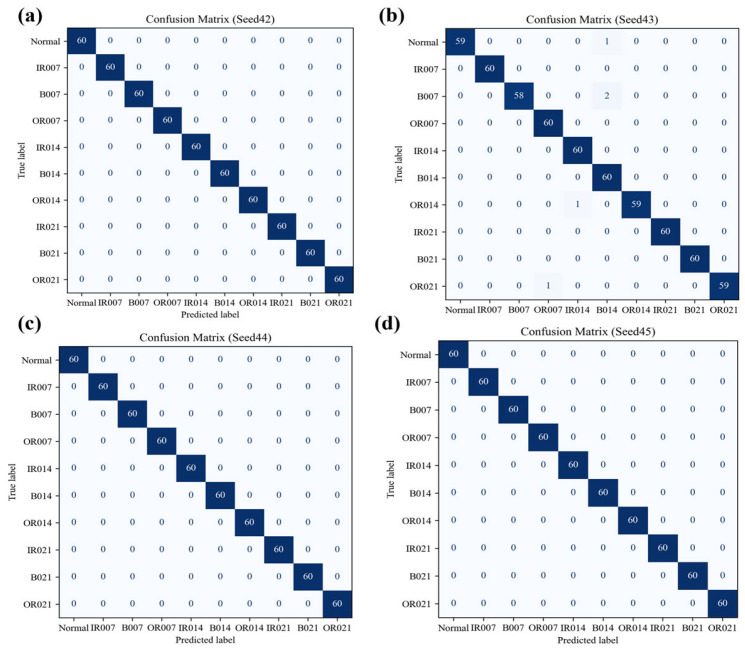
Confusion matrices for the WOA-SVM model on the independent test set under the MCAR5_linear scenario (four random seeds: Seed42, Seed43, Seed44, and Seed45) demonstrate near-perfect classification performance across all categories, with three seeds achieving flawless 100% accuracy across all 600 test samples, while the remaining seed exhibited only minimal misclassification errors exclusively between adjacent classes.

**Figure 16:**
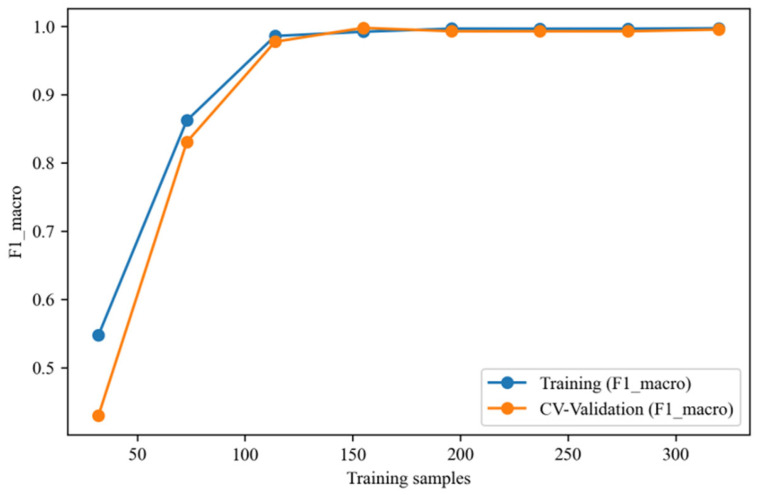
The learning curve of the WOA-SVM model based on the macro-averaged F1-score (macro-F1) under the MCAR5_linear scenario (Seed42) depicts closely converging performance trajectories between the training and cross-validation sets, stabilizing above 99% beyond approximately 120 training samples, demonstrating robust generalization without discernible overfitting indications.

Experimental results demonstrate near-perfect classification performance on the independent test set under MCAR5_linear conditions (Table 12). Confusion matrices across four random seeds (Figure 15) indicate nearly flawless classification, with three seeds achieving 100% accuracy across 600 test samples and the remaining seed showing only minor misclassification between adjacent categories. Learning curves (Figure 16) reveal that after approximately 120 training samples, both training and cross-validation macro-F1 values stabilize above 99%, with minimal divergence between curves indicating model convergence without significant generalization degradation. These findings collectively confirm (1) high consistency on unseen data evidenced by low standard deviations; (2) exclusion of randomness through multiseed validation; and (3) absence of overfitting indicated by congruent training/validation curves. Therefore, the proposed method demonstrates robust generalization capability without significant overfitting risk for the current task.

### 6.5. Practical Implications

Raw signals acquired by field vibration sensors are directly transmitted to edge computing nodes, where edge-layer processing completes signal preprocessing (e.g., normalization and segmentation) and real-time VMD, leveraging VMD’s noise resistance and adaptive bandwidth constraints to rapidly extract IMFs; the features of decomposed IMF components undergo encrypted transmission to factory-level server clusters, where the server-deployed RCMWPE module performs multiscale entropy feature extraction, utilizing the parameter optimization advantages of the WOA-SVM model to achieve high-accuracy centralized fault classification; diagnostic results are pushed in real time via industrial IoT protocols to predictive maintenance platforms, automatically triggering tiered alert mechanisms, generating equipment health assessment reports, and interfacing with work order management systems to provide immediate data support for maintenance decisions. This architecture integrates the real-time capabilities of edge computing with the computational power of cloud computing, ensuring diagnostic accuracy while effectively reducing unplanned downtime and guiding precise maintenance scheduling.

## 7. Discussion

This study innovatively integrates VMD, RCMWPE, and WOA-SVM to establish an integrated multimodal processing framework for bearing fault diagnosis. The methodology effectively suppresses mode aliasing in nonlinear vibration signals through VMD’s adaptive decomposition, while RCMWPE-based multiscale entropy feature extraction constructs a highly discriminative feature space (validated via t-SNE visualization). The WOA algorithm achieves global optimization of SVM penalty parameter *C* and kernel parameter *γ*, delivering superior performance on both PRONOSTIA (96.5% accuracy for four-class classification) and CWRU (99.67% accuracy for ten-class classification) datasets, significantly outperforming conventional approaches. Dual-dataset validation confirms the method’s exceptional robustness and generalizability across variable operating conditions and compound-fault scenarios, establishing a rigorous theoretical framework for intelligent industrial equipment maintenance. Future research will focus on lightweight algorithm optimization to meet real-time diagnostic requirements, enhance adaptability to variable-speed/variable-load conditions, explore hybrid architectures with deep transfer learning, and integrate multiphysics sensing signals (vibration–temperature–current) to develop comprehensive diagnostic systems.

## Figures and Tables

**Table 1:** PRONOSTIA bearing fault dataset.

Number	Simulated Fault Type	Simulated Load Case
1	Healthy Condition	1800 rpm; 1000 N
2	Inner Raceway Fault	1800 rpm; 4000 N
3	Rolling Element Fault	1650 rpm; 4200 N
4	Outer Raceway Fault	1500 rpm; 5000 N

**Table 2:** Central frequencies corresponding to varied modal numbers (PRONOSTIA dataset).

K	Central Frequency				
2	1580	3920			
3	940	1942	2795		
4	695	1750	2645	3875	
5	620	1284	1693	2582	3265

**Table 3:** Cross-correlation coefficients with raw signal across decomposition methods (PRONOSTIA dataset).

Algorithm	IMF1 Cross-Corr	IMF2 Cross-Corr	IMF3 Cross-Corr	IMF4 Cross-Corr
EMD	0.5167	0.3592	0.1723	0.0637
EEMD	0.6537	0.5678	0.4547	0.2235
CEEMD	0.6852	0.6587	0.5187	03867
VMD	0.7323	0.6089	0.6023	0.5853

**Table 4:** CWRU bearing fault classification datasets.

Fault Diameter	Motor Load (HP)	Approx. Motor Speed (rpm)	Inner Race	Ball	Outer Race
0.007”	0	1797	IR007_0	B007_1	OR007@6_0
0.014”	0	1797	IR014_0	B014_1	OR014@6_0
0.021”	0	1797	IR021_0	B021_1	OR021@6_0

**Table 5:** Comparative analysis of cross-correlation coefficients between IMF components extracted by different decomposition methods and original signals in the CWRU dataset.

	EMD	EEMD	CEEDM	VMD
IMF1	0.9413	0.9400	0.9397	0.2753
IMF2	0.1955	0.3434	0.2136	0.1818
IMF3	0.2426	0.2696	0.2256	0.5954
IMF4	0.0738	0.2443	0.1455	0.7415

**Table 6:** Comparison of Classification Accuracy Between SVM and WOA-SVM Based on VMD-RCMWPE Features Under Different Penalty Factor α Values (CWRU 10-Class Noise-Free Data).

α	1000	1500	2000	2500
ACC-WOA	99.50%	99.50%	99.67%	99.17%
ACC	93.00%	94.83%	94.67%	94.50%

**Table 7:** Classification Accuracy Comparison on Four-Category PRONOSTIA Dataset.

Classification Model	Accuracy
WOA-SVM	96.5%
PSO-SVM	92.8%
GA-SVM	91.2%
SVM	88.7%

**Table 8:** Classification accuracy comparison on ten-category CWRU dataset.

Classification Model	Accuracy
WOA-SVM	99.67%
POS-SVM	95.33%
GA-SVM	94.17%
SVM	94.67%

**Table 9:** A comparison of classification accuracy across various SVM Models under different signal-to-noise ratio (SNR) conditions for the ten-class fault diagnosis task on the CWRU dataset.

SNR/dB	SVM	GA-SVM	POS-SVM	WOA-SVM
−5	71.33%	76.17%	77.17%	77.17%
0	82.00%	83.17%	87.67%	95.83%
5	93.00%	93.83%	96.50%	98.83%
10	90.67%	93.17%	97.67%	99.00%
15	93.83%	89.50%	99.17%	99.67%
20	94.33%	94.33%	97.83%	99.00%

**Table 10:** The effects of missing rate and imputation methods under MCAR mechanism on the classification performance of WOA-SVM.

Rate	Imputation	Acc (±SD)	Macro-F1 (±SD)	Kappa (±SD)
5%	ffill	0.993 ± 0.003	0.993 ± 0.003	0.992 ± 0.003
	linear	0.992 ± 0.004	0.992 ± 0.004	0.991 ± 0.004
	spline	0.996 ± 0.004	0.996 ± 0.004	0.996 ± 0.004
10%	ffill	0.989 ± 0.002	0.989 ± 0.002	0.988 ± 0.002
	linear	0.991 ± 0.005	0.991 ± 0.005	0.990 ± 0.006
	spline	0.985 ± 0.013	0.985 ± 0.013	0.984 ± 0.015
20%	ffill	0.987 ± 0.006	0.987 ± 0.006	0.986 ± 0.007
	linear	0.988 ± 0.006	0.988 ± 0.006	0.987 ± 0.006
	spline	0.985 ± 0.009	0.985 ± 0.009	0.984 ± 0.010
30%	ffill	0.969 ± 0.006	0.969 ± 0.006	0.966 ± 0.007
	linear	0.980 ± 0.007	0.981 ± 0.006	0.978 ± 0.007
	spline	0.988 ± 0.005	0.988 ± 0.005	0.987 ± 0.006
40%	ffill	0.945 ± 0.008	0.945 ± 0.008	0.939 ± 0.009
	linear	0.964 ± 0.010	0.964 ± 0.009	0.960 ± 0.011
	spline	0.980 ± 0.006	0.980 ± 0.005	0.978 ± 0.006

**Table 11:** Paired *t*-test analysis of classification performance differences between optimized models and standard SVM baseline.

	*t*	*p*	Statistical Significance
GA-SVM	−6.8124	0.0001	*p* < 0.01
POS-SVM	5.3289	0.0005	*p* < 0.01
WOA-SVM	12.4265	<0.0001	*p* < 0.001

**Table 12:** The WOA-SVM model achieved the following average performance metrics (mean ± standard deviation) on the independent test set under the MCAR5_linear scenario, based on four repeated experiments with distinct random seeds, where MCAR5_linear denotes the random and independent removal of 5% sample values from the original dataset under the missing completely at random (MCAR) mechanism followed by reconstruction via linear interpolation.

Metrics	Mean ± Standard Deviation
Accuracy	0.998 ± 0.004
Macro-F1	0.998 ± 0.004
Micro-F1	0.998 ± 0.004
Kappa	0.998 ± 0.004

## Data Availability

The data are available from the corresponding author on reasonable request.

## Supplementary Materials

The following supporting information can be downloaded at: https://www.mdpi.com/article/10.3390/s25165139/s1, Note S1: Refined Composite Multiscale Weighted Permutation Entropy (RCMWPE); Note S2: t-SNE Dimensionality Reduction. References [44,45,46,47] are cited in the Supplementary Materials.
