# Supplementary material for: Research on Bearing Fault Diagnosis Based on VMD-RCMWPE Feature Extraction and WOA-SVM-Optimized Multidataset Fusion
Source: Sensors (Basel). 2025 Aug 19;25(16):5139. doi: 10.3390/s25165139 (PMC12390204; doi:10.3390/s25165139)
Supplement: Supplementary file 1 [file sensors-25-05139-s001.zip › sensors-3809057-supplementary.pdf]

Article

Research on Bearing Fault Diagnosis Based on VMD-RCMWPE Feature Extraction and WOA-SVM-Optimized Multidataset Fusion

Shouda Wang , Chenglong Wang *, Youwei Lian , and Bin Luo

National Engineering Research Center for Technology and Equipment of Green Coating, Lanzhou Jiaotong University, Lanzhou 730070, PR China

* **Correspondence: Chenglong Wang**

Note S1. Refined Composite Multiscale Weighted Permutation Entropy (RCMWPE)

Regarding feature extraction for fault signals, although various entropy-based metrics (e.g., multiscale entropy, multiscale Shannon entropy, and Rényi entropy) have been extensively studied and applied, these methods frequently encounter limitations when processing non-stationary signals, including pronounced scale sensitivity, inadequate noise robustness, and high sample dependency. In contrast, RCMWPE integrates the time-series sensitivity of permutation entropy, multiscale modeling capability, and a composite weighting mechanism. This synthesis endows RCMWPE with superior dynamic variation perception and enhanced noise immunity. Empirical studies have demonstrated its effectiveness in feature separability and pattern discrimination for mechanical fault diagnosis. Consequently, this study prioritizes RCMWPE as the primary feature extraction methodology, underscoring our focus on robust multiscale feature modeling. Future work will include a systematic comparative analysis between RCMWPE and alternative entropy features to further enhance methodological comprehensiveness.

RCMWPE is a time series complexity quantification method that integrates a multiscale analysis framework, weighted permutation entropy mechanism, and refined composite strategy[1]. This triple-technique coupling significantly enhances the accuracy and noise robustness of complexity measurements. Its key breakthroughs include (1) multiscale analysis capturing scale-dependent features of time series; (2) weighted permutation entropy incorporating amplitude-based weighting to strengthen local feature characterization; and (3) refined composite algorithm optimizing the coarse-graining process to suppress scale calculation biases. This framework enables precise analysis of nonlinear systems' complexity evolution characteristics across multiple scales.

Permutation Entropy (PE)

Permutation entropy (PE) is a symbolic dynamics-based method for quantifying the randomness and complexity of time series. For a time series $X = \{x_1, x_2, \ldots, x_N\}$ of length *N*, the computational procedure of PE involves the following steps:

1. Phase Space Reconstruction: The time series is reconstructed into m-dimensional vector sequences $Y_j = \left[x_j, x_{j+\tau}, \ldots, x_{j+(m-1)\tau}\right]$, where j=1，2，…，N-（m-1），with m being the embedding dimension and t the time delay;
2. Symbolization: Each reconstructed vector Y_j is transformed into an ordinal pattern by arranging its elements in ascending order, yielding an index sequence $\pi_k = \left(r_0, r_1, \ldots, r_{m-1}\right)$, where r_i denotes the position of the Y_j i-th element in the sorted vector;

3. Probability Distribution Calculation: For a given embedding dimension m, there exist m! possible distinct symbol patterns. The occurrence probability $P(\pi_k)$ is computed for each symbolic sequence π_k ;

4. Permutation Entropy Calculation: The permutation entropy of the time series is defined as

$$PE(m,\tau) = -\sum_{k=1}^{m!} P(\pi_k)\ln P(\pi_k) \quad (1)$$

A larger PE value indicates stronger randomness and complexity in the time series, while a smaller PE value corresponds to more regular patterns.

Multiscale Permutation Entropy

Multiscale permutation entropy (MPE) extends the standard permutation entropy (PE) by incorporating a multiscale analysis framework[2]. It constructs time series subsets at different scales through coarse-graining, then computes PE values for each subset to characterize complexity features across scales. The coarse-graining process is defined as follows:

For a scale factor s, the coarse-grained time series $Y_j^{(s)}$ is obtained by

$$Y_j^{(s)} = \frac{1}{s}\sum_{i=(j-1)s+1}^{js} x_i,\ j = 1,2,\ldots,\lfloor N/s \rfloor \quad (2)$$

Then, the permutation entropy is calculated for each coarse-grained time series at scale s to obtain the MPE.

Weighted Permutation Entropy (WPE)

Weighted permutation entropy (WPE) enhances the standard permutation entropy (PE) by incorporating weight factors[3]. It strengthens the characterization of local features and evolutionary trends in time series through unequal contribution weighting of reconstructed vectors. The core mechanism assigns weights based on the variance of elements within each reconstructed vector—higher variance indicates greater information content, thus receiving proportionally larger weight allocation.

Refined Composite Multiscale Weighted Permutation Entropy (RCMWPE)

RCMWPE integrates refined composite multiscale analysis with weighted permutation entropy. Its refined composite multiscale analysis mechanism extends the observation range through non-integer scale coarse-graining, incorporating fractional scale analysis on the basis of integer scales, which significantly improves the characterization accuracy of multiscale features in time series[1].

The computational procedure of RCMWPE is as follows:

1. Refined Composite Coarse-Graining: For a given scale factor s and starting point j (1 ≤ j ≤ s), generate s coarse-grained time series $Y_{j,k}^{(s)}$:

$$Y_{j,k}^{(s)} = \frac{1}{s}\sum_{i=k}^{k+s-1} x_i,\ k = j, j+s,\ldots, j+\left(\lfloor (N-j)/s \rfloor\right)s \quad (3);$$

2. Weighted Permutation Entropy Calculation: Compute the weighted permutation entropy for each coarse-grained time series $Y_{j,k}^{(s)}$;

3. Composite Averaging: The RCMWPE value at each scale is obtained by averaging the weighted permutation entropy across all coarse-grained time series.

RCMWPE demonstrates superior performance in providing more accurate characterization of time series complexity, exhibiting enhanced noise robustness, and

delivering better feature discriminability across different scales. In this study, the RCMWPE parameters are configured as embedding dimension m = 5, maximum scale factor s = 20, time delay τ= 1, and series length N = 2048.

Note S2 t-SNE Dimensionality Reduction

t-SNE is a nonlinear dimensionality reduction method designed for high-dimensional data visualization[4]. Its core mechanism involves converting similarities between high-dimensional data points into probability distributions, then reconstructing corresponding probability distributions in low-dimensional space to map high-dimensional data into 2D/3D space while preserving the topological integrity of both local and global data structures.

The t-SNE computational procedure comprises the following:

High-Dimensional Space Similarity Computation: For any two data points x_i and x_j in high-dimensional space, their similarity $p_{j|i}$ is typically defined using a Gaussian distribution:

$$p_{j|i} = \frac{\exp\left(-\|x_i - x_j\|^2 / 2\sigma_i^2\right)}{\sum_{k\neq i}\exp\left(-\|x_i - x_k\|^2 / 2\sigma_i^2\right)} \qquad (1)$$

where σ_i is the variance of the Gaussian distribution centered at xi, determined via binary search binary search to ensure each data point has identical perplexity. To symmetrize the similarity matrix, the joint probability $p_{ij} = (p_{j|i} + p_{i|j})/(2N)$ is typically employed.

Let $\{y_1, y_2, \cdots, y_N\} \in \mathbb{R}^d$ denote the corresponding low-dimensional embeddings of the original data points. The similarity between mapped points y_i and y_j is defined by a Student's t-distribution with one degree of freedom (equivalent to a Cauchy distribution):

$$q_{ij} = \frac{\left(1 + \|y_i - y_j\|^2\right)^{-1}}{\sum_{k\neq l}\left(1 + \|y_k - y_l\|^2\right)^{-1}} \qquad (2)$$

Here, $\|\cdot\|$ denotes the Euclidean norm in low-dimensional space. The resulting joint probability distribution $Q = \{q_{ij}\}$ ensures $\sum_{i\neq j} q_{ij} = 1$. The heavy-tailed nature of the t-distribution mitigates the crowding problem commonly encountered in low-dimensional projections, enhancing the separation of distinct clusters.

Optimization Objective: The goal of t-SNE is to minimize the Kullback–Leibler divergence (KL divergence) between the high-dimensional and low-dimensional probability distributions:

$$C = KL(P\|Q) = \sum_{i\neq j} p_{ij}\log\frac{p_{ij}}{q_{ij}} \qquad (3)$$

The cost function is optimized via gradient descent, iteratively adjusting the positions of low-dimensional mapped points until convergence. t-SNE plays a pivotal role in the visualization and dimensionality reduction of high-dimensional feature data. It effectively reveals the intrinsic data structure and clustering patterns, providing intuitive insights for subsequent classification tasks.

References

[1] Zhang, Y.P.; Shang, P.J. Refined Composite Multiscale Weighted-Permutation Entropy of Financial Time Series. *Physica A* **2018**, 496, 189-199.

[2] Ouyang, G.X.; Li, J.; Liu, X.Z.; Li, X.L. Dynamic Characteristics of Absence Eeg Recordings with Multiscale Permutation Entropy Analysis. *Epilepsy Res.* **2013**, 104 (3), 246-252.

[3] Fadlallah, B.; Chen, B.D.; Keil, A.; Príncipe, J. Weighted-Permutation Entropy: A Complexity Measure for Time Series Incorporating Amplitude Information. *Phys. Rev. E.* **2013**, 87 (2), 7.
[4] van der Maaten, L.; Hinton, G. Visualizing Data Using T-Sne. *J. Mach. Learn. Res.* **2008**, 9, 2579-2605.
